# Life table of *Myrmeleon fuscus* Yang (Neuroptera: Myrmeleontidae) fed on different natural diets

**DOI:** 10.1093/jisesa/ieag014

**Published:** 2026-03-15

**Authors:** Tingting Zhang, Xiaokang Li, Meng Liu, Lisheng Zhang, Mengqing Wang, Yuyan Li, Zhongjian Shen, Li Zheng, Jianjun Mao

**Affiliations:** Key Laboratory of Natural Enemy Insects of Ministry of Agriculture and Rural Affairs, State Key Laboratory for Biology of Plant Diseases and Insect Pests, Institute of Plant Protection, Chinese Academy of Agricultural Sciences, Beijing, China; School of Advanced Manufacturing, Fuzhou University, Jinjiang, P. R. China; Jinjiang City Fuzhou University Science and Education Park Development Center, Fuzhou University, Jinjiang, P. R. China; Key Laboratory of Natural Enemy Insects of Ministry of Agriculture and Rural Affairs, State Key Laboratory for Biology of Plant Diseases and Insect Pests, Institute of Plant Protection, Chinese Academy of Agricultural Sciences, Beijing, China; School of Advanced Manufacturing, Fuzhou University, Jinjiang, P. R. China; Jinjiang City Fuzhou University Science and Education Park Development Center, Fuzhou University, Jinjiang, P. R. China; Key Laboratory of Natural Enemy Insects of Ministry of Agriculture and Rural Affairs, State Key Laboratory for Biology of Plant Diseases and Insect Pests, Institute of Plant Protection, Chinese Academy of Agricultural Sciences, Beijing, China; School of Advanced Manufacturing, Fuzhou University, Jinjiang, P. R. China; Jinjiang City Fuzhou University Science and Education Park Development Center, Fuzhou University, Jinjiang, P. R. China; Key Laboratory of Natural Enemy Insects of Ministry of Agriculture and Rural Affairs, State Key Laboratory for Biology of Plant Diseases and Insect Pests, Institute of Plant Protection, Chinese Academy of Agricultural Sciences, Beijing, China; Key Laboratory of Animal Biosafety Risk Prevention and Control (North) of Ministry of Agriculture and Rural Affairs, Shanghai Veterinary Research Institute, Chinese Academy of Agricultural Sciences, Shanghai, China; Key Laboratory of Natural Enemy Insects of Ministry of Agriculture and Rural Affairs, State Key Laboratory for Biology of Plant Diseases and Insect Pests, Institute of Plant Protection, Chinese Academy of Agricultural Sciences, Beijing, China; Key Laboratory of Natural Enemy Insects of Ministry of Agriculture and Rural Affairs, State Key Laboratory for Biology of Plant Diseases and Insect Pests, Institute of Plant Protection, Chinese Academy of Agricultural Sciences, Beijing, China; Key Laboratory of Natural Enemy Insects of Ministry of Agriculture and Rural Affairs, State Key Laboratory for Biology of Plant Diseases and Insect Pests, Institute of Plant Protection, Chinese Academy of Agricultural Sciences, Beijing, China; Key Laboratory of Marine Eco-Environmental Science and Technology, First Institute of Oceanography, Ministry of Natural Resources, Qingdao, China; Key Laboratory of Natural Enemy Insects of Ministry of Agriculture and Rural Affairs, State Key Laboratory for Biology of Plant Diseases and Insect Pests, Institute of Plant Protection, Chinese Academy of Agricultural Sciences, Beijing, China

**Keywords:** antlion, rearing, life table, prey

## Abstract

Antlion larvae are sit-and-wait predators that capture arthropods in conical sand traps. The antlion *Myrmeleon fuscus* Yang (Neuroptera: Myrmeleontidae) is a potential biocontrol agent for the red imported fire ant *Solenopsis invicta* Buren (Hymenoptera: Formicidae). In this study, we evaluated the life table of *M. fuscus* when fed on 4 different prey species. The larval stage was shortest when the vetch aphid *Megoura crassicauda* Mordvilko (Hemiptera: Aphididae) was provided as prey for *M. fuscus* larvae. Furthermore, the larval survival rate, pupation rate, pupal weight, and emergence rate of *M. fuscus* fed on *M. crassicauda* were the highest, suggesting that aphids are the most palatable prey for *M. fuscus* larvae. *M. fuscus* adults did not lay eggs when fed with *S*. *invicta* or *M. crassicauda*. They showed a longer oviposition period, higher fecundity, greater intrinsic rate of increase (*r*), higher net reproductive rate (*R*_0_) and greater finite rate of increase (*λ*) and shorter mean generation time (*T*) when fed on *Tenebrio molitor* Linnaeus (Coleoptera: Tenebrionidae) than on *Corcyra cephalonica* Stainton (Lepidoptera: Pyralidae), indicating that *T. molitor* was the most suitable for *M. fuscus* adults. The body weight of *M. fuscus* fed on *T. molitor* larvae was the highest at the third instar. Their body lengths continuously increased from the first instar and peaked at the adult stage. These results contribute to the potential for mass production and application of *M. fuscus.*

## Introduction

The red-imported fire ant, *Solenopsis invicta* Buren, belongs to the order Hymenoptera and family Formicidae. *S*. *invicta* is one of the most devastating insect pests worldwide. It poses serious threats to agriculture, public safety, human health, and ecological security (Zeng et al. 2005). In 2018, *S*. *invicta* spread to more than 390 counties, 15 provinces, and regions in southern China. Currently, *S*. *invicta* is rapidly expanding (Wang et al. 2020, Li et al. 2023).

Management of *S*. *invicta* depends mainly on the use of chemical baits, which effectively suppress its populations. However, the toxicity of chemical pesticides to nontarget organisms and their persistence as environmental residues are indisputable (Li et al. 2018). Biological control is preferred for the long-term management of *S*. *invicta*. Natural enemy insects can either directly kill *S*. *invicta* or indirectly compete with native ants to suppress its populations. Among these natural enemies, the phorid fly *Pseudacteon* has demonstrated potential for controlling *S*. *invicta* (Lawrence and Lloyd 1997, Chen and Fadamiro 2018, Li et al. 2018). However, the parasitism rate of phorid flies is very low, making the eradication of *S*. *invicta* difficult (Calcaterra et al. 2008). To date, the predatory natural enemies of *S. invicta* have rarely been reported.

One of the potential natural enemies of ants are antlions (Neuroptera: Myrmeleontidae), which are predators widely distributed in tropical regions. Myrmeleontidae is a species-rich family within the order Neuroptera. To date, approximately 2,000 extant species belonging to more than 200 genera have been identified worldwide (Devetak et al. 2010, Lu et al. 2019). According to the “Catalog of the World Antlions,” the genus *Myrmeleon* comprises 176 described species (Stange 2004). Ten of these species have been reported from mainland China, including *Myrmeleon fuscus* Yang (Neuroptera: Myrmeleontidae), which is mainly distributed in the central and southern regions (Bao et al. 2009). Antlion larvae are sit-and-wait predators that capture prey using ambush techniques. They constructed funnel-shaped traps in sandy soil and remained buried while waiting for the prey to pass through the soil surface (Abot et al. 2022). They detect the presence of prey at a distance of several centimeters by sensing vibrations generated through prey movement (Devetak et al. 2007, Mencinger-Vracko and Devetak 2008). The behavior and survival of antlions are influenced by both biotic and abiotic environmental factors. Antlions were found to be equally abundant in microhabitats with varying levels of disturbance. However, their density was greater in protected microhabits than in exposed microhabits (Lima 2020). The investment made by antlions in building traps represents a plastic phenotypic response that varies with the surrounding environment. In protected areas, antlions invest more in trap size, which consequently enhances their capture success (Abot et al. 2022). Larval antlions in large traps captured both large and small prey, whereas those in small traps captured only small prey. The trap size and capture success are proportional to the size of antlion larvae (Klokočovnik and Devetak 2013). After a month without food, larval antlions move on average once every 10 days, construct progressively smaller traps, and travel longer distances before establishing a new trap (Heinrich and Heinrich 1984).

Larval antlions are generalist arthropod predators. A large proportion of their diet consists of ants (Topoff 1977). Other arthropods include ticks, beetles, isopods, flies, caterpillars, wasps, lepidopteran larvae, spiders, and mites (Topoff 1977, Crowley and Linton 1999). Analysis of gut contents revealed that many adult Neuroptera are omnivorous or phytophagous. Adult Myrmeleontidae occasionally consume nonanimal foods (New 1991). Larval antlions pass through 3 instars before pupation. They have a remarkable ability to withstand hunger and can survive for up to 3 months without eating. Mature antlion larvae spin silk cocoons before entering the pupal stage, which they remain in for approximately 1 month. Adult antlions are nocturnal predators that are frequently attracted to light (Heinrich and Heinrich 1984, New 1991). They lay eggs in the sand and usually live for about 1 month (Arnett and Gotelli 2001). The life cycle of antlions lasts for 6 months to 2 years, depending on factors such as the availability of food sources, photoperiod, temperature, and metabolic rate (Fisher 1989, Arnett and Gotelli 1999, 2001, Missirian et al. 2006). To date, most studies on antlions have focused on the behavior of larvae in predation and intra- and interspecific competition (Crowley and Linton 1999, Lima 2020, Abot et al. 2022), and some have addressed their energy requirements, fat storage, and tolerance to starvation (Griffiths 1982, 1985, 1991, Lucas 1985, Van Zyl et al. 1997). However, the biology, nutritional requirements, predation spectra, and life tables of adult antlions have rarely been documented.

Recently, the strong predatory ability of *M. fuscus* against *S*. *invicta* has been demonstrated. The third-instar *M. fuscus* larvae had a theoretical maximum predation amount of 47.17 small *S*. *invicta* workers per day. In the field mesocosm experiment, foraging *S. invicta* workers were reduced by 24.43% on the 30th day after release of the third-instar *M. fuscus* larvae (Zhang et al. 2026). These studies demonstrates that *M. fuscus* is a potential biological control agent of *S*. *invicta* (Zhang et al. 2026). However, to date, the biological characteristics of this antlion species remain completely unknown, and substitute prey suitable for mass rearing has not yet been identified. Are the prey that do not naturally occur in the antlions’ habitat palatable to them? To answer this question, in the present study, we compared the effects of several substitute prey species on the life history and reproduction of *M. fuscus* with the aim of identifying suitable prey for the mass production of this natural predator.

## Materials and Methods

### Collection and Rearing of Antlions

*M. fuscus* larvae collected from Shakeng Village, Guangchang County, Fuzhou City, Jiangxi Province, China (26°45′N, 116°18′E) were individually housed in plastic cups (8 cm diameter and 15 cm height). The bottom of the cups was covered with a 5-cm-thick layer of sand with a grain diameter of 0.425 mm. First-instar larvae were fed *Tenebrio molitor* Linnaeus (Coleoptera: Tenebrionidae) once daily; the second- and third-instar larvae were fed twice daily. The pupae were transferred to transparent plastic feeding boxes (33 cm long, 21.7 cm wide, 15.5 cm high) without lids. The bottoms of the boxes were covered with sand similar to that used in cups. A Petri dish containing a cotton ball saturated with water was placed inside each feeding box. Dry branches were provided as habitat for *M. fuscus* adults, which were also supplied with *T*. *molitor*. The masses of prey supplied to *M. fuscus* are shown in Table 1. The feeding box was placed in a cage (45 × 45 × 50 cm^3^). *M. fuscus* culture and prey were maintained at 28 °C, 60% relative humidity under a 14 h:10 h light:dark photoperiod.

**Table 1.** Average daily mass of prey provided to *M. fuscus*

*M. fuscus*	Mass of prey daily offered to *M. fuscus*							
	*S. invicta*		*T. molitor*		*C. cephalonica*		*M. crassicauda*	
	Type	Mass (mg)	Stage	Mass (mg)	Stage	Mass (mg)	Stage	Mass (mg)
**1st instar**	Small workers	1.08	1st instar	1.80	1st instar	1.61	Adult	2.68
**2nd instar**	Small workers	2.15	1st instar	3.59	1st instar	3.25	Adult	5.48
**3rd instar**	Small workers	2.71	1st instar	3.57	1st instar	3.23	Adult	8.14
**Adult**	Small workers	3.78	4-5th instar	51.50	5th instar	25.40	Adult	10.75

### Developmental Duration and Survival of *M. fuscus* Larvae

Freshly laid *M. fuscus* eggs were collected and buried approximately 2 cm deep in sand. An initial population of 30 newly hatched *M. fuscus* larvae was used for each prey treatment. The *M. fuscus* larvae were transferred to plastic cups using a fine brush and reared individually to avoid cannibalism. The bottom of each cup was covered with sand, as described above. The *M. fuscus* larvae were provided with ample prey, including small *S. invicta* workers, *T. molitor* larvae, rice moth *Corcyra cephalonica* Stainton (Lepidoptera: Pyralidae) larvae, and vetch aphid *Megoura crassicauda* Mordvilko (Hemiptera: Aphididae) adults. The masses of the supplied prey are shown in Table 1. The first-instar *M. fuscus* larvae were fed once daily and the second- and third-instar *M. fuscus* larvae were fed twice daily. The prey carcasses were cleaned, and metamorphosis and death were monitored daily. The first-instar stage was defined as the period from hatching to the first ecdysis, the second-instar stage as the period from the first to the second ecdysis, and the third-instar stage as the period from the second ecdysis to pupation.

### Pupation and Emergence

After pupation, cocoon size and mass were measured. Pupal size was recorded as the diameter of the cocoons. The pupation rate was calculated as the ratio of successfully pupated larvae to the initial number of larvae. Cocoons were poured into a sieve, and the surrounding sand was removed by gentle shaking prior to weighing on a precision scale (XP205; Mettler Toledo, Switzerland) with an accuracy of 0.0001 g. After weighing, the cocoons were transferred to feeding boxes without lids. Cotton balls saturated with water and dry branches were provided. The feeding boxes were then placed in mesh cages. Adult emergence was monitored daily.

### Reproduction and Adult Life Span

Male and female *M. fuscus* were paired in plastic boxes (25.2 × 17.4 × 9.3 cm^3^) with mesh lids. The 4 prey species were provided twice a day. The heads of *T. molitor* and *C. cephalonica* larvae were removed to expose their body fluids as their big size and hard exoskeleton makes it difficult for the antlion adults to prey on them. Inside the boxes, the 2 prey items were held by hand, attracting the antlions to fly over and feed on them. Feeding lasted for 2 min. *S. invicta* small workers and *M. crassicauda* adults were dropped to the bottom of the box. The number of eggs (F1 generation, offspring of male and female pairs) was counted daily. The lifetime fecundity (number of eggs oviposited by each female throughout the adult stage), preoviposition period (between emergence and the first day of oviposition), and oviposition period (between the first and last days of oviposition) were recorded. To measure the hatching rate, 10 F_1_ eggs were randomly collected and neonates were counted. The hatching rate was evaluated across 10 replicates. Adult lifespan is expressed as the period from emergence to death.

### Body Length and Weight of *M. fuscus* Fed on *T. molitor*

The body length and weight of *M. fuscus* fed on *T. molitor* were measured in separate experiments. To estimate the size of *M. fuscus* larvae and adults, body length (from the tip of the head to the end of the abdomen) was measured using calipers. Larvae and adults were weighed on a precision scale (XP205, Mettler Toledo, Switzerland). The sizes and masses of the first-, second-, and third-instar *M. fuscus* larvae were measured 8, 10, and 15 days after hatching. *M. fuscus* adults were measured on day 2 after eclosion. Ten individuals were examined for each parameter at each developmental stage.

### Data Analysis

The demographic characteristics of *M. fuscus* that fed on *S. invicta* workers, *T. molitor* larvae, *C*. *cephalonica* larvae, and adult *M. crassicauda* were analyzed using the TWOSEX-MSChart program (Chi 2022) based on the age-stage, two-sex life table theory (Chi et al. 2020). Age-specific survival rate (*l_x_*), age-specific fecundity (*m_x_*), net maternity (*l_x_m_x_*), intrinsic rate of increase (*r*), net reproductive rate (*R*_0_), finite rate of increase (*λ*) and mean generation time (*T*) were calculated as previously described (Chi and Liu 1985). The differences in these life parameters among different prey treatments were analyzed by paired bootstrap test (*B* = 1,000) based on 95% CIs of the difference. These are expressed as follows:


$$R_{0}=\sum_{x=0}^{\infty }l_{x}m_{x}$$

$$\sum_{x=0}^{\infty }e^{-rm(x+1)}l\mathrm{xmx}=1$$

$$\lambda =e^{r}$$

$$T=\frac{\mathrm{In}Ro}{r}$$

Homogeneity of variance was confirmed before testing the means. Differences in the duration of the larval stage among different prey treatments, and body weight and body length among different developmental stages were determined using 1-way analysis of variance (ANOVA Least Significant Difference). Differences in first-, second-, and third-instar stages, pupal stage, adult lifespan, survival rate, pupation rate, pupal weight, pupal diameter, and emergence rate among the 4 prey treatments were determined using the nonparametric Kruskal–Wallis test. Differences in the oviposition period and lifetime fecundity between the *T. molitor* and *C. cephalonica* groups were determined using the Student *t* test. Differences in the preoviposition period and egg hatching rate between the *T. molitor* and *C. cephalonica* groups were analyzed using the nonparametric Mann–Whitney test.

## Results

### Duration of Larval and Pupal Stages and Adult Lifespan

**Fig. 1.**
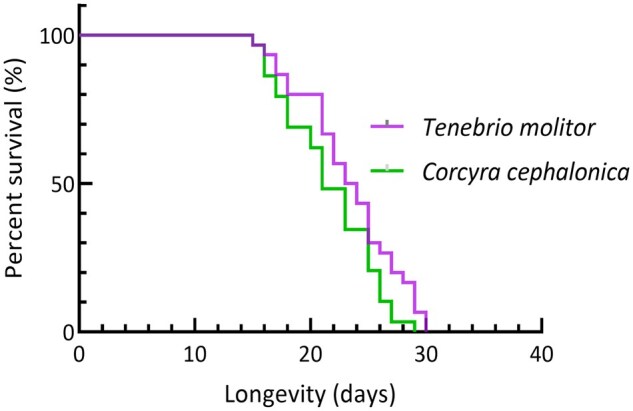
Life expectancy of *Myrmeleon fuscus* adults fed on different prey.

The *M. fuscus* first-instar stage (*H* = 69.872, *df *= 3, *P *< 0.01), second-instar stage (*H* = 69.421, *df *= 3, *P *< 0.01), and third-instar stage (*H* = 40.051, *df *= 3, *P *< 0.01) were significantly affected by the prey. The larval stage of *M. fuscus* that fed on *S. invicta* workers was significantly longer than that of *M. fuscus* that fed on *T. molitor* larvae, *C*. *cephalonica*, and *M. crassicauda* (*F*_3,113_=132.133, *P *< 0.001). The larval stage of *M. fuscus* feeding on *M. crassicauda* adults was the shortest, suggesting a rapid development rate. No significant differences were observed in the duration of pupal stage among the four prey treatments, suggesting that pupal development was not significantly affected by prey (*H* = 3.578, *df *= 3, *P *= 0.311). *M. fuscus* adults did not consume *T. molitor* and *C*. *cephalonica* larvae. They died of starvation at early adult stage. The *M. fuscus* adult lifespan was significantly longer when reared on these 2 prey species than on *S. invicta* workers and *M. crassicauda* adults (*H* = 89.253, *df *= 3, *P *< 0.01) (Table 2, Fig. 1).

**Table 2.** Developmental periods and adult lifespans of *M. fuscus* fed on different preys

Prey	Developmental period (d)					
	1st instar	2nd instar	3rd instar	Larval stage	Pupal stage	Adult lifespan
***S. invicta***	13.48 ± 0.34a	14.67 ± 0.33a	18.41 ± 0.41a	46.56 ± 0.64a	26.92 ± 0.53a	4.22 ± 0.19b
***T. molitor***	9.27 ± 0.32b	12.46 ± 0.22b	16.00 ± 0.26bc	37.73 ± 0.47c	25.90 ± 0.53a	23.33 ± 0.79a
***C. cephalonica***	9.40 ± 0.28b	13.83 ± 0.25a	16.27 ± 0.34b	39.50 ± 0.45b	26.06 ± 0.42a	21.73 ± 0.72a
***M. crassicauda***	7.66 ± 0.19c	10.20 ± 0.24c	14.73 ± 0.26c	32.60 ± 0.42d	26.43 ± 0.40a	3.83 ± 0.18b

*M. fuscus* adults did not consume *T. molitor* and *C*. *cephalonica* larvae. They died of starvation at early adult stage. Means ± SE of larval stage followed by different letters within a column indicate significant differences (*n* = 30, *P *< 0.05). Differences in the entire larval stage among the different prey species were determined using 1-way ANOVA. Differences in first instar, second instar, and third instar stages, pupal stage, and adult lifespan among the prey were determined using the nonparametric Kruskal–Wallis test.

### Survival Rate

The survival rates of *M. fuscus* at different larval stages were all above 90% when fed on *S. invicta* workers and reached 100% when fed on *T. molitor* larvae, *C*. *cephalonica* larvae, and adult *M. crassicauda.* The survival rates at the first-instar stage (*H* = 3.000, *df *= 3, *P *= 0.392), second-instar stage (*H* = 6.600, *df *= 3, *P *= 0.086), third-instar stage (*H* = 0.000, *df *= 3, *P *= 1.000), and throughout the larval stage (*H* = 6.545, *df *= 3, *P *= 0.088) did not differ significantly among the four prey treatments (Table 3).

**Table 3.** Survival rates of *M. fuscus* at different ages

Prey	Initial population	Survival rates at different stages (%)			
		1st instar	2nd instar	3rd instar	Larval stage
***S. invicta***	30	96.67 ± 3.33a	93.33 ± 3.33a	100.00 ± 0.00a	90.00 ± 5.77a
***T. molitor***	30	100.00 ± 0.00a	100.00 ± 0.00a	100.00 ± 0.00a	100.00 ± 0.00a
***C. cephalonica***	30	100.00 ± 0.00a	100.00 ± 0.00a	100.00 ± 0.00a	100.00 ± 0.00a
***M. crassicauda***	30	100.00 ± 0.00a	100.00 ± 0.00a	100.00 ± 0.00a	100.00 ± 0.00a

Means ± SE followed by the same letters within a column are not statistically different (Kruskal–Wallis test, *P *< 0.05).

### Pupation Parameters

There were no significant differences in pupal mass among the 4 prey treatments (*H* = 4.211, *df *= 3, *P *= 0.240). The *M. fuscus* pupation rate was not significantly affected by the prey (*H* = 6.600, *df *= 3, *P *= 0.086). The differences in pupal diameters among the 4 prey treatments were not significant (*H* = 5.050, *df *= 3, *P *= 0.168). The emergence rate was not significantly affected by the prey (*H* = 3.000, *df *= 3, *P *= 0.392) (Table 4).

**Table 4.** Pupation parameters of *M. fuscus* fed on different preys

Prey	Initial population	Pupation rate (%)	Pupal weight (mg)	Diameter (mm)	Emergence rate (%)
***S. invicta***	30	93.33 ± 3.33a	320.43 ± 6.50a	8.91 ± 0.09a	96.67 ± 3.33a
***T. molitor***	30	100a	327.95 ± 6.55a	9.16 ± 0.06a	100a
***C. cephalonica***	30	100a	321.54 ± 5.78a	9.02 ± 0.06a	100a
***M. crassicauda***	30	100a	335.08 ± 5.40a	9.11 ± 0.06a	100a

The data in the table are expressed as mean ± SE, and same letters within a column indicate no significant differences (Kruskal–Wallis test, *P *< 0.05).

### Reproduction

Female adults supplied with *S. invicta* workers and *M. crassicauda* adults did not oviposit, as they neither attacked nor consumed these 2 prey species. The preoviposition period (*U* = 70,000, *Z* = −0.789, *P *= 0.479) and egg hatchability (*U* = 40.500, Z = −0.744, *P *= 0.481) did not differ significantly between the *T. molitor* and *C*. *cephalonica* treatments; however, the oviposition period (*P *= 0.045) and lifetime fecundity (*P<* 0.001) did (Table 5).

**Table 5.** Reproductive characteristics of *M. fuscus* fed on different preys

Prey	Preoviposition period (d)	Oviposition period (d)	Fecundity (ind.)	F1 hatchability (%)
***S. invicta***	—	—	—	—
***T. molitor***	8.23 ± 0.23a	17.00 ± 0.75a	14.38 ± 0.73a	87.00 ± 0.50a
***C. cephalonica***	8.54 ± 0.27a	15.00 ± 0.58b	10.39 ± 0.59b	83.00 ± 0.37a
***M. crassicauda***	—	—	—	—

*M. fuscus* females that fed on *S. invicta* and *M. crassicauda* did not oviposit, because they did not eat these 2 prey species. Means ± SE of fecundity (*n* = 13) and oviposition period (*n* = 13) followed by different letters within a column indicate significant differences determined by Student *t* test (*P *< 0.05). Means of the preoviposition period (*n* = 13) and egg hatching rate (*n* = 10) followed by different letters within a column indicate significant differences as determined by the Mann–Whitney test (*P *< 0.05).

### Population Parameters

The net reproductive rate (*R*_0_) was zero, and the intrinsic rate of increase (*r*), finite rate of increase (*λ*) and mean generation time (*T*) were not provided by the TWOSEX-MSChart program in the *S. invicta* and *M. crassicauda* treatments, as *M. fuscus* supplied with these 2 prey did not lay eggs. The 4 population parameters did not differ significantly between *T. molitor* and *C*. *cephalonica* treatments (Table 6).

**Table 6.** Life table parameters of *M. fuscus* fed on different preys

Prey	Intrinsic rate of increase (*r*) (d^−1^)	Net reproductive rate (*R*_0_)	Finite rate of increase (*λ*) (d^−1^)	Mean generation time (*T*) (d)
***S. invicta***	—	0	—	—
***T. molitor***	0.021 ± 0.003a	6.233 ± 1.363a	1.020 ± 0.0033a	88.851 ± 0.911a
***C. cephalonica***	0.017 ± 0.003a	4.500 ± 0.967a	1.017 ± 0.003a	89.278 ± 1.237a
***M. crassicauda***	—	0	—	—

SEs were estimated using 1,000 bootstraps and the means were compared using a paired bootstrap test. Means ± SE followed by different letters within a column indicate significant differences (*n* = 30, *P *< 0.05).

### Size of *M. fuscus* Fed on *T. molitor*

**Fig. 2.**
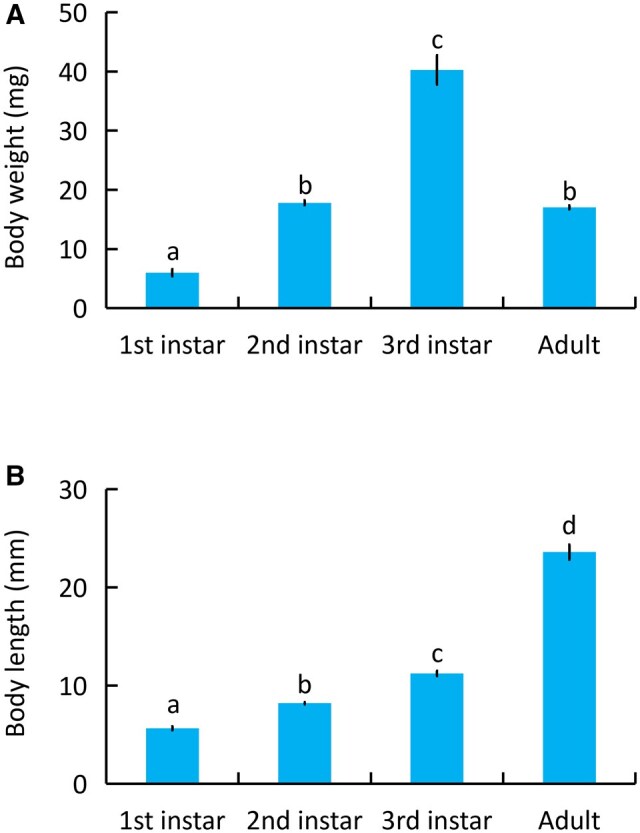
Body weight and length of *M. fuscus* fed on *T. molitor*. A) Body weight. B) Body length. Bars represent means ± SE of 10 replicates. Different letters above the bars indicate significant difference (*n* = 10, 1-way ANOVA followed by LSD, *P *< 0.05).

The body weight of *M. fuscus* fed on *T. molitor* increased rapidly at the larval stage and declined at the adult stage. However, the body length increased continuously with increasing age and peaked at the adult stage. The average body weights of the first instar, second instar, third instar, and adults were 5.98 ± 0.59, 17.81 ± 0.38, 40.26 ± 2.50, and 17.01 ± 0.32 mg, respectively (*F*_3,36_=121.12, *P<*0.001) (Fig. 2A). The mean body lengths of the first instar, second instar, third instar, and adults were 5.64 ± 0.19, 8.21 ± 0.10, 11.24 ± 0.26, and 23.60 ± 0.76 mm, respectively (*F*_3,36_=457.67, *P<*0.001) (Fig. 2B).

## Discussion

Augmentative biological control relies on mass production of high-quality natural enemies. An ample and cost-effective diet is fundamental for mass production of generalist predators (Sahayaraj 2001, Riddick 2009). The diets of generalist predators can be classified into 2 major categories based on their sources: natural prey and artificial diets, each with its distinct advantages and disadvantages. Currently, biocontrol producers usually raise natural enemies on their natural prey or host, which, in turn, maintained on their host plants. Unlike natural prey, an artificial diet does not involve maintenance of the 3 trophic levels (Riddick 2009). However, developing a palatable artificial diet that meets the nutritional requirements of predators and supports the continuous production of offspring is a challenging task (Riddick 2009). Furthermore, to date, natural prey suitable for the mass rearing of antlion species, especially antlion adults, has not been exploited. Therefore, in the present study, we investigated the life table of a Myrmeleontidae species, when fed on 4 prey species from different orders, with the aim of identifying palatable natural prey suitable for its mass production.

Although antlion larvae feed on ant species such as the red imported fire ant and leaf-cutting ants (*Atta* spp.) (Missirian et al. 2006, Zhang et al. 2026), these prey are not suitable for the mass rearing of antlions. This is because indoor rearing and maintenance of ant colonies are difficult and sufficient supply of ants are not available in the market. Therefore, we selected 3 nonant insects, *M. crassicauda*, *C. cephalonica*, and *T. molitor* as prey of *M. fuscus*, using *S*. *invicta* as a reference. We argue that whether antlions attack the 3 nonant species in the wild is irrelevant, as the goal of the present study was to screen for the best prey for indoor mass production of *M. fuscus*. The vetch aphid, *M. crassicauda* can be raised on a large scale using broad bean seedlings as hosts. *C. cephalonica* and *T. molitor* are in ample supply in the market at low cost. Therefore, if these prey prove palatable, the mass production of *M. fuscus* could be achieved cost-effectively. As expected, *M. fuscus* showed the longest larval stage when fed on *S*. *invicta* (Table 2). This indicates that the nonant prey species are more palatable to *M. fuscus* larvae than *S*. *invicta*, because nutritious diet can shorten larval developmental period (Omkar and Srivastava 2003, Arshad et al. 2021). Most important of all, *M. fuscus* did not consume *S*. *invicta* and died of starvation, implying that *S*. *invicta* is not palatable to *M. fuscus* adults. The possible reason is that antlion adults have different digestive physiology from larvae and cannot metabolize the venom proteinaceous and alkaloidal toxins contained in ants (Touchard et al. 2016). It is worth mentioning that the manual feeding method for the *M. fuscus* adults may cause deviations in their behavior, as we found that they did not consume *T. molitor* and *C. cephalonica* larvae placed at the bottom of the box. We cannot completely rule out the possibility that the *M. fuscus* adults were attracted by the manual feeding action, rather than by the exposed body fluid of the prey.

We found that *M. fuscus* larvae and adults have different prey preferences by comparing the effects of prey on the demographic parameters of *M. fuscus* larvae and adults. Among the 3 nonant prey species that do not naturally occur in the habitat of *M. fuscus*, *M. crassicauda* proved to be most palatable to *M. fuscus* larvae. This conclusion is supported by the short larval stage and high larval survival rate, pupation rate, pupal weight, and emergence rate of *M. fuscus* larvae fed with *M. crassicauda*. However, among the 3 nonant prey species, *T. molitor* was the most palatable to *M. fuscus*. This is because the adult lifespan and oviposition period were the longest, and fecundity, intrinsic rate of increase (*r*), net reproductive rate (*R*_0_), and finite rate of increase (*λ*) were the highest when *T. molitor* was provided to *M. fuscus* adults. *R*_0_ is an important parameter that is closely related to population growth and fecundity (Sayyed et al. 2008). The maximum *R*_0_ value of *M. fuscus* adults fed on *T. molitor* suggests that this prey promoted rapid development and consequently led to a high reproductive rate and a long oviposition period for the predator (Amer et al. 2018, Azher et al. 2019).

The pupation parameters of *M. fuscus* were not significantly affected by prey. This is consistent with the results of another antlion species, *Myrmeleon brasiliensis* Navás (Neuroptera, Myrmeleontidae). When *M. brasiliensis* larvae were fed different prey, including leaf-cutting ants (*Atta* spp.), fruit fly larvae (*Anastrepha* spp. and *Ceratitis capitata*), and a mixed diet (*Atta* spp. plus fruit flies), neither the pupal period nor the pupal size was significantly influenced by the prey type (Missirian et al. 2006). Similar results indicated that natural prey with different palatability had no discernible effects on the pupation of the two antlion species.

*M. fuscus* adults died soon after being supplied with *S*. *invicta* workers and *M. crassicauda* adults because they did not consume the prey. This outcome may not occur under natural conditions, where a variety of arthropods in the natural habitat are available as potential prey for *M. fuscus* adults. *M. fuscus* adults fed on *C. cephalonica* and *T. molitor* showed normal reproductive performance, demonstrating that these 2 prey species successfully sustained the reproduction of *M. fuscus* adults. However, the lifetime fecundity (14.38) of *M. fuscus* adults that fed on *T. molitor* was relatively low, suggesting that this prey was not palatable enough for *M. fuscus* adults. To address this limitation, further studies are required to identify prey species that can better sustain adult reproduction in *M. fuscus*. Another solution is to provide multiple prey species that can improve food quality and increase the reproductive output of polyphagous predators (Topoff 1977). For example, *M. brasillensis* antlions showed the shortest developmental duration when fed a mixed diet (leaf-cutting ants plus fruit fly larvae), which was related to a more comprehensive composition and better nutritional quality of the diet. Finally, supplementation with plant components, such as pollen, may contribute to the reproduction of antlions (Missirian et al. 2006).

This study indicated that the vetch aphid *M. crassicauda* was the most suitable for mass rearing of *M. fuscus* larvae, followed by *T. molitor*. Although *T. molitor* is not as palatable as *M. crassicauda* for *M. fuscus* larvae, it is cost-effective and has an abundant supply in the market. *T. molitor* was the most palatable species for *M. fuscus* adults. Considering the palatability and availability of the prey tested, we conclude that *T. molitor* is the most suitable species for mass production of *M. fuscus* throughout its life stage. Based on this conclusion, we measured the body length and weight of *M. fuscus* fed on *T. molitor*. The *M. fuscus* demographic parameters obtained will contribute to the screening of more palatable prey and development of artificial diets.
